# Nurses’ attitudes toward addressing sexual health in their professional practice within cancer care: a Danish cross-sectional study

**DOI:** 10.1007/s00520-025-09969-7

**Published:** 2025-10-14

**Authors:** Louise Bregnhøj Mortensen, Line Lønbro Boisen, Bell Møller, Luise Sinding Nygaard, Anna Cecilie Jørgensen, Clara Rosengaard Groth, Kristina Holmegaard Nørskov

**Affiliations:** 1 Department of Oncology, Sygehus Lillebælt, Vejle, Denmark; 2 Multidisciplinary Pain Center, Regional Hospital Silkeborg, Silkeborg, Denmark; 3 Copenhagen Center for Cancer and Health, The Municipality of Copenhagen, Copenhagen, Denmark; 4 Department of Oncology, Herlev and Gentofte Hospital, Herlev, Denmark; 5 Department of Urology, Herlev and Gentofte Hospital, Herlev, Denmark; 6 Sanos, Søborg, Denmark; 7 Department of Haematology, Zealand University Hospital, Roskilde, Denmark

**Keywords:** Attitudes, Sexual health, Cancer care

## Abstract

**Purpose:**

To investigate Danish nurses specializing in cancer care in terms of their attitudes toward addressing sexual health in their professional practice.

**Methods:**

A nationwide cross-sectional survey was conducted in June–August 2024 among Danish registered nurses working in cancer care. The validated Health Professionals’ Attitudes Towards Addressing Sexual Health (PA-SH-D) questionnaire was used to assess attitudes across four domains: comfort, patient encounters, colleagues, and education. Data was analyzed using descriptive statistics and linear regression.

**Results:**

A total of 538 nurses completed the survey. Most respondents were female (97.6%) with a mean age of 45.9 years and an average seniority of 18.8 years. The nurses felt comfortable, to some extent, about informing (37.8%), initiating (44.1), and discussing (45%) sexual health issues with patients, although around half of the nurses (45.3%) would not set aside time to deal with sexual health issues. Knowledge about where to seek information and to participate in sexual health training in the past year was significantly associated with higher total PA-SH-D scores (respectively *p* < 0.001 and *p* < 0.05). Seniority was positively associated with preparedness (*β* = 0.18 per year, *p* < 0.05). Cultural sensitivities, patient discomfort, and lack of time were frequently addressed as barriers.

**Conclusion:**

While many Danish oncology and hematology nurses report feeling comfortable discussing sexual health, significant barriers remain. Training and access to relevant resources are associated with greater perceived preparedness. Our findings highlight a critical need for ongoing education and institutional support to integrate sexual health as a standard component of oncology and hematology nursing care.

**Supplementary Information:**

The online version contains supplementary material available at 10.1007/s00520-025-09969-7.

## Introduction

Sexual health, a fundamental aspect of overall well-being and quality of life, is an integral aspect of one’s personality and identity [1]. A cancer diagnosis and its treatment may affect sexual health, potentially becoming a significant challenge during survivorship [2–5]. Receiving information and support concerning sexual changes after cancer, however, is associated with positive psychological and sexual adjustment [6–8]. Research nevertheless suggests that cancer patients have unmet sexual health needs that are underrecognized and not routinely addressed in the healthcare system [9, 10].

It is widely recognized that sexual dysfunction is particularly prevalent among cancer patients and can manifest as reduced sexual desire and arousal, with women experiencing pain during intercourse and having difficulty achieving orgasm [11–15]. For women, this may include dyspareunia, vaginal dryness, insufficient lubrication, vaginal atrophy, and stenosis [5, 11, 16]. Conversely, men experience challenges with erectile dysfunction, ejaculatory dysfunction, hypogonadism, and increased skin sensitivity [16–18]. These sexual health challenges vary across genders and are influenced by the specific cancer diagnosis, stage, and treatment type [15]. Additionally, individual factors, such as age and comorbidities, also affect the nature and extent of these difficulties, highlighting the need for tailored interventions to address the diverse sexual health needs of patients with cancer [13, 19, 20]. Previous research has mainly focused on sexuality in cancers that directly affect the sexual or reproductive organs, such as prostate, testicular, breast, and gynecological cancer [15, 21]. However, the evidence highlights that cancer also has a significant impact on the sexual well-being of patients with non-reproductive cancers [22, 23]. Unlike many other symptoms or late effects of cancer treatment, sexual problems or reduced sexual health can persist or potentially worsen for several years after treatment ends [24].

Despite the significance of sexual health challenges and the prevalence of sexual dysfunction in the context of oncology and hematology, it often remains inadequately addressed in cancer care [21, 23, 25, 26]. Common barriers that healthcare professionals often report are time constraints, insufficient training, physical environment, and uncertainty about their professional role [10, 27, 28]. Specifically, a resent scoping review by Alqaisi et al. (2025) highlights the importance of education, ongoing support, and cultural competence in strengthening oncology nurses’ capacity to address the sexual health concerns of cancer patients [26]. Similarly, a Danish qualitative study emphasized that sexuality remains taboo, with patients and healthcare professionals deliberately avoiding discussing sexuality-related issues [29]. In line with this, a systematic review (2019) exploring the nurses’ skills in delivering sexual healthcare to patients with cancer emphasized that nurse-led provision of sexual healthcare remains ineffective and challenging, mainly due to lack of professional confidence in dealing with sexual issues and the nurses’ assumptions toward sexuality [30]. Consequently, patients may not receive the support they need to manage the sexual health challenges their diagnosis and treatment pose.

Despite the importance of addressing sexual health in patients with cancer and the evidence emphasizing sexual dysfunction as an unmet need in this population, few studies have investigated nurses’ attitudes regarding sexual health issues. In this context, attitudes refer to nurses’ beliefs, feelings, and predispositions toward addressing sexual health in clinical practice, including their perceived importance of the topic, level of comfort, and willingness to engage in related discussions with patients. To ensure that nurses are adequately prepared to address sexual health concerns in cancer patients, knowledge on nurses’ knowledge, attitudes, and practices can contribute to identifying knowledge gaps in education and training, ultimately improving the quality of care and patient outcomes. In this study, we focus specifically on nurses’ attitudes, as measured by a validated tool designed to assess healthcare professionals’ attitudes toward addressing sexual health. The aim of this study was to investigate Danish nurses specializing in cancer care in terms of their attitudes toward addressing sexual health in their professional practice.

## Material and methods

This Danish nationwide exploratory cross-sectional study was conducted in accordance with the Strengthening the Reporting of Observational Studies in Epidemiology (STROBE) statement guidelines for reporting observational studies [31]. Based at Zealand University Hospital, Roskilde, Denmark, the study was conducted in collaboration with the national sexuality and cancer group under the auspices of the Danish Professional Society for Cancer Nurses.

### Participants and recruitment

All Danish registered nurses were invited to participate if > 18 years and affiliated with a clinical practice in either an oncological or hematological department in Denmark. Eligible participants were identified through the Danish Professional Society for Cancer Nurses and the chief nurses at the various oncological and hematological departments in Denmark. Eligible participants received an e-mail in June 2024 with an information letter about the study and a direct link to a self-administered online questionnaire called Health Professionals’ Attitudes Towards Addressing Sexual Health (PA-SH-D). A reminder was sent to non-responders in August 2024.

### Data collection

Demographic and occupational data, including gender, age, and seniority, were collected from the participants. The PA-SH-D questionnaire assessed the healthcare professionals’ attitudes toward incorporating sexual health into their professional practice [32]. PA-SH-D is a validated, modified Danish version of the original Swedish questionnaire called Students’ Attitudes towards Addressing Sexual Health which showed strong internal consistency, with a Cronbach’s alpha of 0.89 (95% CI 0.85) and acceptable floor and ceiling effects ranging from 0.0 to 13.7% [33]. In our study, we saw similar strong internal consistency with a Cronbach’s alpha of 0.882 (95% CI [0.865, 0.897]). As in [33], we considered floor- and ceiling-effects to be present if > 15% scored respectively the highest or lowest item score. Floor effects were present in 37% of the questions while ceiling effects were present in only 5%. PA-SH-D comprises 22 items, measured on a five-point Likert scale (disagree, partially disagree, partially agree, agree, completely agree). The responses “agree/completely agree” are considered positive for positively charged items; for negatively charged items, the responses “disagree/partially disagree” are considered as expressing a positive attitude. PA-SH-D comprises four domains: feeling comfortable (items 1–9), patient encounters (items 10–15), colleagues (items 16–18), and education (items 19–22). PA-SH-D has a possible total score of 22 to 110, and three response patterns: uncomfortable and unprepared (score 22–56), comfortable and prepared in some situations (score 57–79), and comfortable and well prepared to work with sexual health in their future profession (score 80–110).

### Ethics

The Danish Data Protection Agency (p-2024–16714) approved the study. All participants were provided with written study information, informed that participation was voluntary, and assured that any identifying information would be anonymized and protected.

### Statistical analysis

Data analysis was conducted using R version 4.4.1 (R Core Team, 2024). Participant characteristics and responses to PA-SH-D items were summarized as counts and percentages for categorical variables and mean (SD) for continuous variables. The total PA-SH-D and domain scores were calculated as the sum of responses, with domain scores normalized to a 0–100 scale in accordance with PA-SH-D recommendations, to account for varying numbers of items per domain [32, 33]. Descriptive statistics were based on a sample size of 538 respondents, while regression analysis was performed on 530 respondents after excluding cases with nonsensible or incomplete responses. Categories with few respondents were excluded to preserve anonymity and reduce uncertainty in the model. No variable had missing values exceeding 4.5%, and 21% of respondents did not answer one or more items (which was 12% for PA-SH-D items). Continuous variables were imputed using the median, and categorical variables were imputed using the mode to prevent bias in total and domain-specific scores. The final analysis was performed using a linear additive regression model with a dependent variable total PA-SH-D score and the independent variables of seniority, age, participation in training on sexual health within the past year, and knowledge of where to seek sexual health information. No significant interactions or non-linear relationships were found. To ensure proper control of the regression model, diagnostics were performed to assess linearity, normality of residuals, homoscedasticity, and multicollinearity among independent variables. The relationships between the independent variables and the dependent variable were examined using a type II analysis of variance (ANOVA) with F-tests. This method assesses the effect of each variable by testing whether removing it from the model significantly reduces the model’s ability to explain variation in the dependent variable. *p* values < 5% were considered statistically significant.

## Results

Of the 1429 nurses approached, 538 (37.6%) consented to participate, completed the assessment, and are included in the analyses. The study population mean age was 45.9 years (range of 24–68), most were female (97.6%) performing clinical work with patients (87.2%) (Table 1). The majority worked with breast (14.1%), gastrointestinal, liver, pancreas, and bile ducts (13.0%) cancer. Level of seniority was 18.8 years (SD 11.7), ranging from newly qualified to 44 years of experience. More than half of the responders (56.2%) had a sexology resource person on staff, and 68% could refer their patients to sexual counseling at the hospital or in their department. The majority (84.4%) also had access to patient information on cancer and sexuality at their department. Finally, 59.4% had not participated in training on sexual health and sexual issues among patients within the past year, and many (44.1%) did not know where to find information on sexual health and sexual issues.

**Table 1:** Demographic and clinical characteristics

Characteristic	Total sample (*N* = 538)
Sex, *n* (%)	
Female	525 (97.6)
Male	6 (1.1)
Unknown, *n* (%)	7 (1.3)
Age, mean (SD)	45.9 (11.5)
Range	24–68
Age categories, *n* (%)	
18–39	177 (32.9)
40–65	349 (64.9)
65 or older	4 (0.7)
Unknown	8 (1.5)
Seniority	
Mean (SD)	18.8 (11.7)
Range	0–44
Unknown, *n* (%)	24 (4.5)
Seniority categories, *n* (%)	
0–5	83 (15.4)
6–10	85 (15.8)
11–20	123 (22.9)
21–30	126 (23.4)
> 31	97 (18.0)
Unknown, *n* (%)	24 (4.5)
Specialization	
Hematology	120 (10.8)
Breast cancer	157 (14.1)
Lung, head, and neck cancer	133 (12.0)
Gastrointestinal cancer, liver, pancreas, and bile ducts	144 (13.0)
Gynecologic cancer	99 (8.9)
Brain cancer	58 (5.2)
Kidney and urological cancer	69 (6.2)
Skin cancer	58 (5.2)
Osteosarcoma and soft tissue sarcoma	45 (4.1)
Rare cancers	25 (2.3)
Radiotherapy	80 (7.2)
Palliative care unit	66 (5.9)
Clinical research unit	13 (1.2)
Phase 1 and 2/experimental treatment	1 (0.1)
Other	22 (2.0)
Unknown	20 (1.8)

### Attitudes toward addressing sexual health

**Fig. 1:**
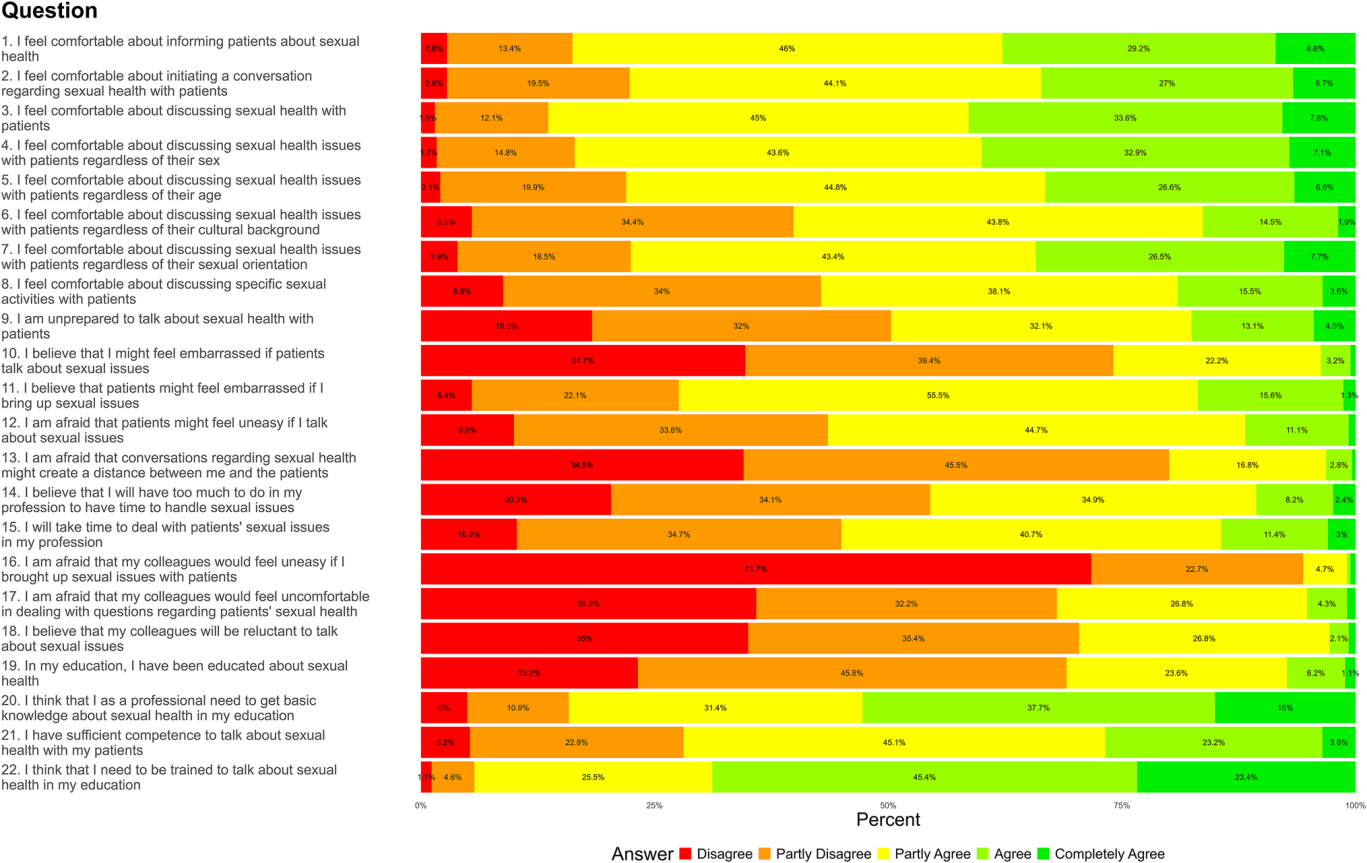
Danish nurses attitudes toward addressing sexual health

The distribution of the total PA-SH-D score between the participants was: comfortable and unprepared (2.8%), comfortable and prepared in some situations (68.4%), and comfortable and well prepared to work with sexual health in their future profession (28.8%). The majority of nurses felt, to some extent, comfortable informing, initiating, and discussing sexual health issues with patients (Fig. 1). However, this became less comfortable for many in terms of the patients’ cultural background and discussing specific activities. Some agreed or partly agreed (25.9%) that they would feel embarrassed if patients asked about sexual issues. Simultaneously, many thought that the patients would feel embarrassed (72.5%) or would feel uneasy talking about sexual issues (46.5%). Half of the participants would not set aside time to deal with these problems (45.3%). Despite these challenges, nearly all agreed that training in sexual health communication should be included in nursing education, although only about half felt they currently possessed sufficient skills. We calculated the raw domain scores as the sum of scores for the items in each domain, while the adjusted domain scores normalize these to a 0–100 scale to account for differences in the number of items per domain (Table 2). Domain 3, colleagues, stands out with a particularly high mean score, indicating that respondents had stronger opinions about items involving their colleagues.

**Table 2:** Domains and total score of health professionals’ attitudes towards addressing sexual health

Core type	Feeling comfortable	Patient encounter	Colleagues	Education	Total score
Raw	28.3 (6.7)	20.9 (3.3)	12.6 (2)	12.5 (2.2)	74.3 (10.5)
Adjusted (%)	62.8 (14.9)	69.8 (11.1)	84.3 (13)	62.4 (10.8)	67.6 (9.5)

### Regression analysis

Regression analyses were conducted using a linear additive model, where the dependent variable was the total PA-SH-D score, and the independent variables were seniority, age, knowledge of where to seek information, and participation in training on sexual health within the past year (Supplementary Material). We found that the relationship between seniority and the total score appears linear. No significant interaction between seniority, training, and knowledge was found.

Respondents who did not have knowledge of where to seek information on sexual health in their specialty scored significantly lower on the total score compared to those who did. Since level 1 represents “Yes” (had knowledge) and level 2 represents “No” (did not have knowledge), the regression coefficient indicates that not having knowledge reduces the total score significantly by 5.38 on average. Respondents who did not receive training on sexual health in the past year scored significantly lower on the total score compared to those who did. Since level 1 represents “Yes” (received training) and level 2 represents “No” (did not receive training), the regression coefficient indicates that not receiving training reduces the total score significantly by 2.12 points on average. For every year of seniority, the total score increases significantly by 0.18 points on average, highlighting a positive relationship between work experience and total score. The intercept with a value of 77.18 points represent the expected total score for the reference group, i.e., a female who has received training within the past year and has knowledge on where to seek for information. Age and gender did not have a significant effect on the total PA-SH-D score on a 5% significance level.

## Discussion

### Discussion of findings

This cross-sectional study is the first to report data on the attitudes of Danish nurses in cancer care to discuss sexual health issues with patients. The findings highlight that, although nurses felt comfortable discussing sexual health issues in their professional practice, many still avoided addressing the topic and had concerns that patients might feel embarrassed or uneasy. Possessing knowledge on where to seek information and undergo training on sexual health positively impacted nurses’ level of comfort and preparedness in addressing sexual health issues. The findings emphasize the importance of providing ongoing training and resources for nurses in cancer care to ensure that they feel comfortable and prepared to address sexual health issues with their patients.

This study underscores the critical role that both knowledge and training play in enhancing the ability of nurses to address sexual health issues within cancer care. The findings indicate that nurses who were aware of where to seek information and those who had received training on sexual health felt more comfortable and better prepared to discuss sensitive topics with their patients. This finding aligns with Krouwell et al., who conducted a large cross-sectional study (*n* = 433) that found that one of the main barriers to openly discussing sexual function was lack of training, and the majority (72.9%) of the participants wanted to acquire additional training [34]. Similar to our findings, the participants in their study with more self-reported knowledge discussed sexual function more often [34]. However, their findings contradict a quasi-experimental study examining attitudes, practices, and perceived barriers regarding a sexual health education intervention among oncology professionals that found no changes in frequency of discussing sexual health or in taking the initiative to discuss the topic [35]. This could be due to perceived barriers among the nurses, including unclear expectations regarding the individual nurse’s skill level, or the fact that not all of them wish to become experts in discussing sexual health issues. A systematic review by Albers et al. found that face-to-face, skill-based interventions increase the comfort level of the nurses in terms of engaging in discussion on the topic [21]. This finding emphasizes the importance of providing targeted education and resources in oncology and hematology nursing to foster a supportive and skills-based approach to sexual health, which is often overlooked in routine cancer care.

A qualitative study exploring sexual health communication among nurses further emphasized that nurses lack knowledge of sexual health during their education [36]. In our study, the majority agreed that training in sexual health communication and sexual dysfunction should be included in nursing education. Our findings align with a previous scoping review by Alqaisi et al. (2025) which underscore a clear need for formal, multifaceted education in sexual healthcare for oncology nurses [26]. Integrating sexual health into nursing education is essential for equipping nurses with the necessary knowledge and skills required to address sexual health issues confidently and competently. Furthermore, as studies consistently show, when nurses feel more knowledgeable and trained in sexual health and communication regarding sexual health issues, they are more likely to initiate conversations about sexual health, reducing the stigma and discomfort surrounding these discussions [25].

Several studies highlight that healthcare professionals acknowledge that sexual health counseling falls within their profession, yet they admit to not routinely addressing or informing patients about the sexual side effects of treatment [34]. Consistent with previous research, we found that many of the nurses avoid addressing sexual health issues, which may partly be due to concerns about patient discomfort or cultural sensitivities. This aligns with a Danish qualitative study examining how patients experience sexuality as a taboo in the healthcare system, the findings highlighting that some patients who initiate conversations about their sexuality feel rejected, and that those who are reluctant to discuss sexuality find that healthcare professionals also do not bring up the topic [29]. This two-way taboo and mutual hesitance to communicate about sexual health requires greater attention in clinical practice as it hinders the provision of comprehensive care, which should encompass all aspects of a patient’s well-being, including sexual health, especially in patients undergoing cancer treatment, where sexual health issues are particularly relevant but often overlooked [34].

Sexual norms play a significant role in how sexual health is addressed in healthcare settings and in reinforcing this taboo [37]. The norm that primarily associates sex with young people in permanent and monogamous heterosexual relationships can have various influential effects [38]. Norms in the healthcare system also imply that sexual health is only relevant to discuss when treatment has a curative compared to a life-prolonging intent, especially regarding palliative care [34]. A qualitative study exploring factors that influence healthcare professionals’ conversations about sexual health highlights that sex and age differences between the patient and the healthcare professional may prevent either party from introducing the topic [28]. Similarly, our findings highlight that a patient’s age or cultural background can influence their level of comfort. These norms create barriers that prevent open communication about sexual health. Overcoming these obstacles requires a shift in both societal and healthcare norms to foster an inclusive and comprehensive approach to sexual health in patients with cancer, regardless of treatment type or stage of disease, age, sex, or sexual orientation. Therefore, future research should explore strategies for overcoming these barriers, including the incorporation of cultural skills training in sexual health education for nurses and ensuring that an approach to sexual health based on a broad conceptual framework is consistently integrated into patient care plans.

Our study found that years of experience as a nurse was significantly associated with comfort and preparedness in addressing sexual issues with patients as every year of seniority significantly increased the total PA-SH-D score. Conversely, a systematic review examining the prevalence and predictors associated with sexual health communication found that a greater number of years of experience was significantly associated with greater prevalence of communication about sexual concerns [25]. Being older, which may be confounded with years of experience, was also associated with greater prevalence of sexual health discussions with patients [25]. Importantly, these results underscore that seniority alone does not make nurses comfortable about or prepared to initiate sexual conversations. Although experience may contribute to overall clinical competence, it does not necessarily address the specific skills needed to navigate sensitive topics like sexual health. This aspect highlights the importance of incorporating comprehensive sexual health education into nursing programs in clinical practice to provide ongoing professional development opportunities. Nurses with specialized training in sexual health are more likely to feel confident and competent when discussing sexual health concerns with patients, regardless of their years of experience [21, 25].

Notably, even though the majority of nurses reported having access to a clinician for referral, many still cited a lack of time as a barrier to addressing sexual health concerns directly. This suggests that structural and organizational constraints, such as workload and time pressure, may limit nurses’ ability to engage in sexual health discussions, despite the presence of referral options. Addressing these barriers is essential for improving the integration of sexual health into routine nursing care. Moreover, institutional factors and workplace culture play a significant role in shaping nurses’ readiness to engage in sexual health conversations. In environments where sexual health is not prioritized or where organizational support is lacking, even nurses with positive attitudes toward sexual health may hesitate or feel unprepared to address the topics. Organizational support, including access to resources, guidelines, and collaboration with multidisciplinary teams, is essential for fostering a supportive environment where nurses feel encouraged and equipped to address sexual health concerns [34].

### Discussion of methods

One of the strengths of our study is the broad nationwide sample of nurses specializing in cancer care, which potentially increased the representativeness and generalizability of our findings. Moreover, we used a questionnaire specifically developed to assess attitudes toward addressing sexual health that was validated among healthcare professionals and in a Danish context [32]. A limitation is the cross-sectional design, which prevents the establishment of a temporal association between exposure and outcome. Therefore, any temporal associations should be interpreted with caution. In addition, the low response rate may undermine the validity of the findings due to response bias, as individuals with stronger opinions or more direct experiences with sexual health in cancer care may have been more inclined to participate. This limitation also reduces the generalizability of the results to the broader population of cancer patients. As a result, the study may underestimate nurses’ attitudes toward discussing sexual health in their professional practice. Finally, while the study was conducted across Denmark, it is important to recognize that the findings may not be generalizable to other countries or healthcare systems, where cultural and organizational factors may differ. Despite these limitations, we believe our study offers valuable insight into Danish nurses’ attitudes to address sexual health in their professional practice, highlighting an oft-overlooked aspect of supportive care.

## Conclusion

In conclusion, this study highlights the attitudes of Danish nurses in cancer care regarding sexual health discussions with patients. While most nurses reported feeling comfortable addressing sexual health in their professional practice, many still avoided initiating conversations about it, and had concerns about patient discomfort or cultural sensitivities. The study reveals that nurses who had knowledge about where to seek information and those who had received recent training on sexual health felt more prepared and comfortable about discussing such issues with their patients. The findings underscore the importance of ongoing sexual health training and education for nurses, particularly within oncology and hematology settings. Despite the general level of comfort participants reported, significant barriers, such as patient embarrassment, cultural differences, and lack of institutional support, were identified. These challenges emphasize the need to address sexual health as a core component of nursing education and professional development, ensuring that nurses are equipped with the necessary skills and resources to navigate sensitive discussions effectively. Ongoing training can be offered through regular workshops, seminars, and continuing education programs. Institutions can support nurses by fostering open dialogue, providing access to resources, and establishing policies that prioritize sexual health as a key aspect of holistic patient care.

## Supplementary Information

Below is the link to the electronic supplementary material.

Supplementary Material 1 (PNG 1.14 MB)

## Data Availability

No datasets were generated or analyzed during the current study.

## Declarations

### Clinical trial registration number

Not relevant.
